# Application of left ventricular endomyocardial biopsy in the diagnosis of mitochondrial cardiomyopathy: a case report

**DOI:** 10.1186/s12872-023-03373-x

**Published:** 2023-07-04

**Authors:** Chuangsen Fang, Ming Lan

**Affiliations:** 1 Peking University Fifth School of Clinical Medicine, Beijing, China; 2 Department of Cardiology, Beijing Hospital, National Center of Gerontology; Institute of Geriatric Medicine, Chinese Academy of Medical Sciences, Beijing, P. R. China

**Keywords:** Mitochondrial cardiomyopathy, Endomyocardial biopsy, Heart failure

## Abstract

**Background:**

The clinical features of mitochondrial cardiomyopathy (MCM) are diverse. It can present as hypertrophic cardiomyopathy or dilated cardiomyopathy. The diagnosis of MCM is challenging and usually based on biopsy.

**Case presentation:**

The 30-year-old man was admitted to hospital due to dyspnea for 1 month and edema of both lower extremities for 1 week. Echocardiography suggested a whole heart enlargement, a whole heart diminished function. Renal impairment and diabetes were observed. Coronary angiography showed single-vessel disease (90% stenosis in the ostium of a small marginal branch). Left ventricular endomyocardial biopsy was performed.

**Conclusion:**

Myocardial histopathology demonstrated a large number of abnormal mitochondrial accumulation, so the diagnosis was considered as mitochondrial cardiomyopathy.

## Background

Mitochondrial disease is a group of specific diseases caused by dysfunction of the mitochondrial energy synthesis system [1, 2]. Abnormal mitochondrial number, structure or function leading to abnormal myocardial energy metabolism, and the clinical manifestation of cardiomyopathy is called mitochondrial cardiomyopathy (MCM).

## Case presentation

A 30-year-old man presented with a history of dyspnea for 2 months and edema of both lower extremities for 1 week without precipitating factors such as infection or alcohol abuse. The patient did not show improvement in the above symptoms after receiving standard-dose furosemide diuretic therapy. There was no history of seizure, previous use of medication, diabetes mellitus, hypertension, smoking or cardiac disease. He had no family history of a similar complaint.

Physical examination upon admission: temperature(T) 37.1 °C, pulse(P) 100 beats/min, respiration(R) 18 beats/min, blood pressure (BP) 145/95 mmHg, no wet rales in lungs, neat heart rate, 100 beats/min, A2 > P2, no murmur in the auscultation area of the valve. There were no apparently positive signs in abdomen. Mild edema of both lower extremities was found.

Laboratory examination: blood routine: WBC 12.60*10^9^/L; blood biochemistry: GLU 7.5 mmol/L, CRE 103 μmol/L, UREA 5.94 mmol/L, normal liver function and electrolytes; no obvious abnormality in coagulation function; myocardial injury marker: cTnI 0.06 ng/mL, Myo 25.0 ng/mL, CK-MB 2.6 ng/mL; BNP: 957.94 pg/mL. His NT-pro-BNP was marked elevated at 4075 pg/mL. urine routine: protein 0.5 g/L; 24 h urine protein quantitation 0.15 g/24 h, 24 h urine sodium 304 mmol/24 h, protein quantitative determination of 189.3 mg/L, N-acetyl-β-D glycosaminidase 13U/L. Fasting venous blood glucose was 7.5 mmol/L, and glycated hemoglobin was 7.6%.

Electrocardiogram suggested sinus rhythm, left ventricular hypertrophy, and changed non-specific T wave. Doppler echocardiography showed left ventricular end-diastolic anteroposterior diameter: 48 mm, left ventricular end-diastolic diameter: 64 mm, right atrial diameter: 47 mm, right ventricular end-diastolic anteroposterior diameter: 32 mm, septal thickness: 9 mm, left ventricular posterior wall thickness: 9 mm. Mild mitral regurgitation and tricuspid regurgitation with left ventricular systolic dysfunction was observed (left ventricular ejection fraction, 25%).

Admission diagnosis was heart failure with possible dilated cardiomyopathy. Magnetic resonance imaging (MRI) was terminated, because the patient was pale and sweaty during the examination. Transradial coronary angiography (CAG) illustrated no abnormalities in LM, LAD, or LCX, 25% stenosis in the middle of RCA, 90% stenosis in the sharp margin, and superior coronary in the right coronary, thus diagnosing coronary atherosclerosis heart disease, single-vessel disease, right-handed dominant type. Left ventricular angiography revealed that the left ventricular total systolic function was reduced, and no abnormal wall motion was observed, LVEDP 20-25 mmHg, LVEF 28.5%.

**Fig. 1:**
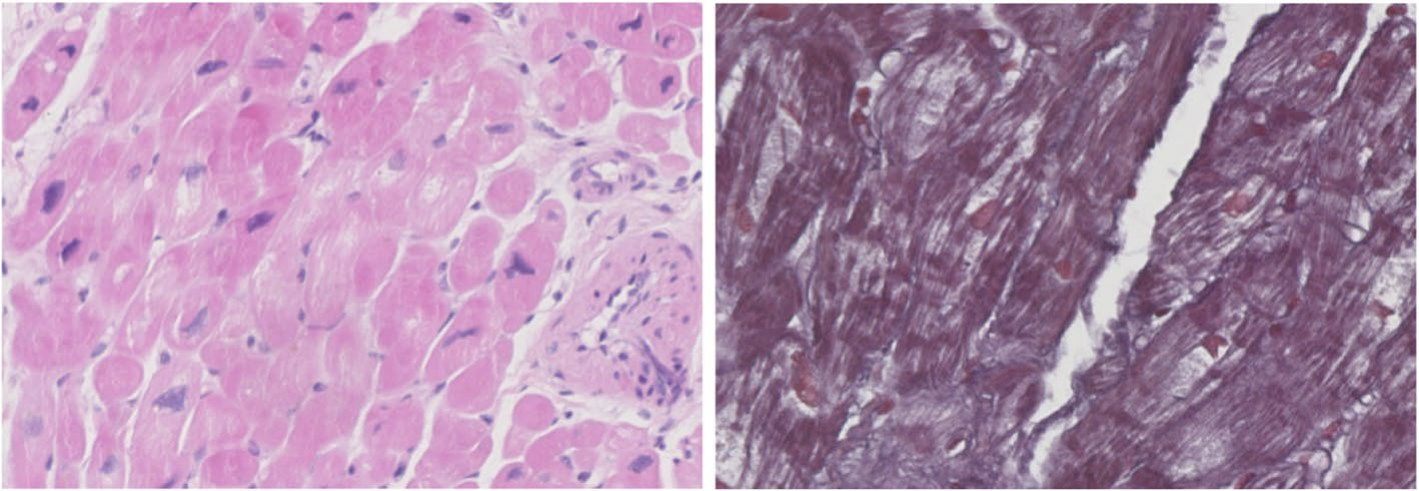
Under light microscopy, worm like changes and nuclear atypia could be observed with cardiomyocytes

**Fig. 2:**
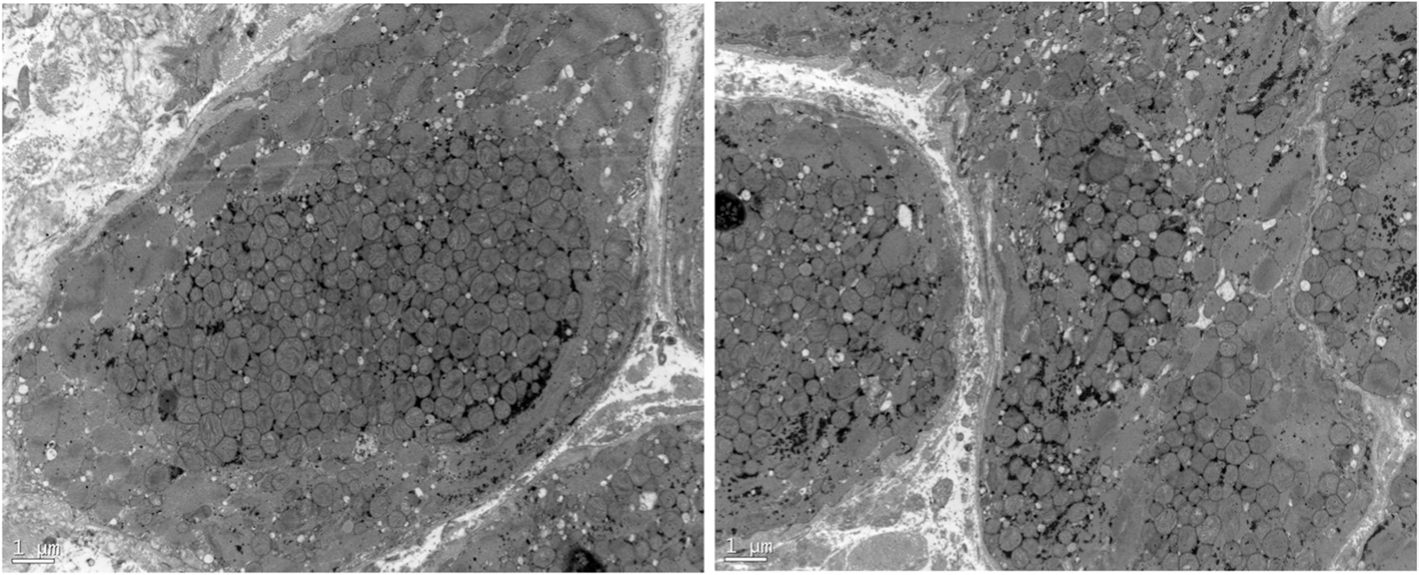
Under electron microscopy, a significant accumulation of mitochondria and interstitial infiltration could be observed with cardiomyocytes

Left ventricular endocardial myocardial biopsy through the same radial artery suggested that degenerative changes occurred in the cardiomyocytes, and extensively "worm-like" changes could be observed. (a large number of abnormal mitochondrial accumulation areas under electron microscopy). The enlargement and nuclear atypia could be observed in cardiomyocytes (Fig. 1 Light micrographs of a left ventricular endomyocardial biopsy specimen). Transmission electron microscopy showed that the myocardial cell structure was disordered with part of the interstitial infiltration, and a large number of abnormal mitochondrial accumulated, so the diagnosis of mitochondrial cardiomyopathy was considered. (Figure 2 Electron microscopy of a left ventricular endomyocardial biopsy specimen). After treatment with diuretic, ACEI, β-blocker, aldosterone receptor antagonists, dyspnea and lower extremity edema disappeared. With one-month follow-up after surgery, no obvious discomfort was complaint.

Daily oral medical therapy was administered strictly according to our instructions after discharge. The symptoms faded and did not recur. Two years follow-up after discharge demonstrated that NT-pro BNP was 65 pg/mL, and echocardiography showed normal ejection fraction (Table 1).

**Table 1:** The progression of the echocardiographic data and the examination findings

	On admission	After treatment	Post admission after 1 month
LAd(mm)	48	43	42
LVEDd(mm)	64	70	66
RAd(mm)	47	38	38
RVEDd(mm)	32	31	29
IVSTd(mm)	10	9	11
PWTd(mm)	10	9	10
EF(%)	25	29	29
NT-pro BNP(pg/mL)	2541	1474	1222
Chest X-ray		(-)	

*LAd* Left atrial diameter, *LVEDd* Left ventricular end diastolic diameter, *RAd* Right atrial diameter, *RVEDd* Right ventricular end diastolic diameter, *IVSTd* Interventricular septum thickness diameter, *PWTd* Posterior left ventricular wall thickness diameter, *EF* Ejection function, *NT-proBNP* NT-pro brain natriuretic peptide

## Discussion

According to Kisler [3], the prevalence of neonatal mitochondrial abnormalities is about 1/7634, and the prevalence of mitochondrial disease in a lifetime is about 1 in 5000. Because a large number of mitochondrial diseases have not been reported, and the disease is clinically and genetically heterogeneity, so accurate morbidity is difficult to estimate. Another study [4] showed that the minimum birth prevalence rate was 6.2/100000, and the prevalence of adult mitochondrial disease caused by mitochondrial DNA (mtDNA) and nuclear genome pathogenic mutations was about 1/4300. Gorman [5] reported that the incidence of mtDNA mutations is approximately 20/100,000, and the incidence of mitochondrial disease in adults that causes clinically significant symptoms is approximately 2.9/100000 in this population.

The possible molecular genetic mechanisms leading to mitochondrial cardiomyopathy are mitochondrial RNA gene point mutations, D-loop gene mutations in the regulatory region, and mtDNA deletion. Anthony [6] has reported tRNA mutations such as 3243A → G, 4269A → G associated with MCM. With the accumulation of mtDNA deletion, respiratory chain function degradation, and free radical-mediated damage, mitochondrial function is gradually reduced [7–9]. An increase in the rate of mtDNA mutations or a decrease in the concentration of intracellular antioxidants will accelerate this process [10].

MCM often involves multiple organs, which can lead to abnormal myocardial structure and function, manifesting as hypertrophic cardiomyopathy, dilated cardiomyopathy, arrhythmia, and left ventricular myocardial insufficiency. Other systemic damage manifests as muscle weakness, liver and kidney dysfunction, diabetes or thyroid dysfunction.

Lev [11] proposes cardiomyopathy if any of the following exists, considering MCM: [1] found respiratory chain enzymes in muscle, fibroblasts or platelets; [2]mutation or deletion of mitochondrial DNA; [3]found broken red fiber in myocardial Gomori staining, or found cytochrome C oxidase staining decreased; [4] a large number of abnormal mitochondrial accumulations were found in electron microscopy; [5] mitochondrial diseases have been confirmed in first-degree relatives. Meyers [12] presents the primary and secondary criteria for the diagnosis of mitochondrial cardiomyopathy in terms of clinical manifestations, histology, enzymology, function, molecular level, and metabolic levels (Table 2).

**Table 2:** Dignostic criteria for mitochondrial disorders

	**Major Diagnostic Criteria**	**Minor Diagnostic Criteria**
Clinical	Mitochondrial syndrome or involvement of any 3 of the following systems: neurologic, muscular, cardiac, renal, nutritional, hepatic, endocrine, hematologic, otologic, ophthalmologic, dermatologic, or dysmorphic; exacerbations; family history of mtDNA mutations; or exclusion of alternate diagnosis	Symptoms compatible with an RC defect
Histologic	> 2% ragged red fibers in skeletal muscle	1%–2% ragged red fibers in a patient 30 to 50 years of age, any ragged red fibers in a patient < 30 years of age, > 2% subsarcolemmal mitochondrial accumulations in a patient < 16 years of age, or widespread electron microscopic abnormalities in any tissue
Enzymologic	> 2% COX-negative fibers in a patient < 50 years of age, > 5% COX-negative fibers in a patient ≥ 50 years of age, < 20% activity of any RC complex in a tissue, < 30% activity of any RC complex in a cell line, or < 30% activity of the same RC complex activity in ≥ 2 tissues	Antibody-based demonstration of a defect in RC complex expression, 20%–30% activity of any RC complex in a tissue, 30%–40% activity of any RC complex in a cell line, or 30%–40% activity of the same RC complex activity in ≥ 2 tissues
Functional	Fibroblast ATP synthesis rates > 3 SD units below the mean	Fibroblast ATP synthesis rates 2 to 3 SD units below the mean, or fibroblasts unable to grow on media with glucose replaced by galactose
Molecular	Identification of an nDNA or mtDNA mutation of undisputed pathogenicity	Identification of an nDNA or mtDNA mutation of probable pathogenicity
Metabolic	-	One or more metabolic indicators of impaired RC function

Reproduced with permission from: Bernier FP, Boneh A, Dennett X, Chow CW, Cleary MA, Thorburn DR. Diagnostic criteria for respiratory chain disorders in adults and children. Neurology 2002;59(9):1406–11.25

*ATP* Adenosine triphosphate, *COX* Cytochrome oxidase, *mtDNA* mitochondrial DNA, *nDNA* nuclear DNA, *RC* Respiratory chain

Definitive = 2 major criteria, or 1 major criterion plus 2 minor criteria; probable = 1 major plus 1 minor, or 3 minor; possible = 1 major, or 2 minor, 1 of which must be clinical

The patient, a young man who was physically healthy, was admitted to the hospital with whole heart failure. Echocardiography suggested a whole heart enlargement and the ejection fraction was only 25%. According to coronary angiography, we diagnosed coronary atherosclerotic heart disease, a single-vessel disease, but the coronary lesions couldn’t explain the patient's whole heart enlargement and severe cardiac insufficiency. It was also accompanied by kidney damage and diabetes. Left ventricular endocardial myocardial biopsy revealed that the cardiomyocytes were extensively "worm-like" changes, which manifested a large number of abnormal mitochondrial accumulation areas under the electron microscope. According to the diagnostic criteria proposed by Lev, in the myocardial ultrastructure of the cardiomyopathy patients, there was a large amount of abnormal mitochondrial accumulation, and MCM should be considered. According to the diagnostic criteria proposed by Meyers, the patient had multiple systemic involvements such as myocardial, renal damage and diabetes. Abnormal mitochondrial accumulation could be seen under electron microscope, which matched one major criterion and one secondary standard, and might be MCM. At this point, the patient's diagnosis was clear, but unfortunately, the patient refused to conduct any further examinations in enzymology, functional science, or molecular science, so we couldn’t obtain more clinical data. Myocardial biopsy is an important breakthrough in the diagnosis of MCM. In other words, obtaining myocardial histopathology and electron microscopy results plays a decisive role in the diagnosis of MCM. A study involving 755 myocardial biopsies and 6371 myocardial biopsy specimens demonstrated that the incidence of severe complications was similar between left ventricular biopsy and right ventricular biopsy (0.64% vs. 0.82%). The incidence of minor complications was lower in the left ventricular biopsy group (0.64–2.89% vs. 2.24–5.10%). The left ventricular biopsy had a higher rate of histopathological diagnostic yield (64.5–74.8% vs. 55.8–57.1%) [13]. Another study involving 4221 patients who underwent EMB (1153 LV EMBs, 672 RV EMBs and 2369 both LV and RV EMBs) showed the overall risk of major complications was remarkably low. (0.33% vs. 0.45%). LV samples revealed diagnostic clues in 96.3% of cases (*n* = 2307) versus 71.4% (*n* = 1711) in RV tissue (*p* < 0.001). In these two trials, although statistical significance was not achieved, the safety profile of left ventricular endomyocardial biopsy appears to have greater potential. This could be further validated through trials with an expanded sample size. In terms of efficacy, left ventricular endomyocardial biopsy provides more comprehensive pathological diagnostic clues, resulting in a higher net value [14].

## Conclusion

This case report manifests that if a young patient, admitted with whole heart failure, has multiple organs dysfunction and clinical features of cardiopathy, mitochondrial cardiomyopathy should be considered. A myocardial biopsy can be performed to determine the type of cardiomyopathy. A Left ventricular endocardial myocardial biopsy helps to improve the diagnostic rate of MCM and is safer. The transradial approach used in these patients also facilitates myocardial biopsy.

## Data Availability

The datasets supporting the conclusions of this article are included within the manuscript.

## References

[1] Gerald P, Chinnery PF (2013). Diagnosis and treatment of mitochondrial myopathies. Ann Med.

[2] Yilmaz A (2010). Comparative Evaluation of Left and Right Ventricular Endomyocardial Biopsy. Circulation.

[3] Kisler JE, Whittaker RG, Mcfarland R (2010). Mitochondrial diseases in childhood: a clinical approach to investigation and management. Dev Med Child Neurol.

[4] Lightowlers RN, Taylor RW, Turnbull DM (2015). Mutations causing mitochondrial disease: What is new and what challenges remain?. Science.

[5] Gorman GS (2015). Prevalence of nuclear and mitochondrial DNA mutations related to adult mitochondrial disease. Ann Neurol.

[6] Anthony HV (2012). S. Mitochondrial diseases. Lancet.

[7] Yevgenya K (2006). Mitochondrial DNA deletions are abundant and cause functional impairment in aged human substantia nigra neurons. Nat Genet.

[8] Spinazzola A (2011). Mitochondrial DNA mutations and depletion in pediatric medicine. Semin Fetal Neonatal Med.

[9] Sato M, Sato K (2013). Maternal inheritance of mitochondrial DNA by diverse mechanisms to eliminate paternal mitochondrial DNA. Biochem Biophys Acta.

[10] Aleksandra T (2004). Premature ageing in mice expressing defective mitochondrial DNA polymerase. Nature.

[11] Lev D (2004). Clinical Presentations of Mitochondrial Cardiomyopathies. Pediatr Cardiol.

[12] Meyers DE, Basha HI, Koenig MK, et al. Mitochondrial cardiomyopathy: pathophysiology, diagnosis, and management. Tex Heart Inst J. 2013;40(4):385-94. PMC3783139 24082366

[13] Yilmaz A (2010). Comparative evaluation of left and right ventricular endomyocardial biopsy: differences in complication rate and diagnostic performance. Circulation.

[14] Chimenti C, Frustaci A (2013). Contribution and risks of left ventricular endomyocardial biopsy in patients with cardiomyopathies: a retrospective study over a 28-year period. Circulation.

